# The effect of massage on feeding intolerance in preterm infants: a systematic review and meta-analysis study

**DOI:** 10.1186/s13052-020-0818-4

**Published:** 2020-04-23

**Authors:** Leila Seiiedi-Biarag, Mojgan Mirghafourvand

**Affiliations:** 1 Department of Midwifery, School of Nursing and Midwifery, Tabriz University of Medical Sciences, Tabriz, Iran; 2 Midwifery Department, Social Determinants of Health Research Center, Tabriz University of Medical Sciences, Tabriz, Iran

**Keywords:** Massage, Feeding intolerance, Preterm infants

## Abstract

**Background:**

Feeding intolerance in premature infants is one of the main causes of their long-term hospitalization in NICUs. Massage therapy is a cost-effective intervention that has a positive impact on the health of infants and their parents. This systematic review investigates the effect of massage on feeding intolerance in preterm infants.

**Methods:**

A search was carried out in English databases including Medline (via PubMed), Scopus, Cochrane Library, Google Scholar, Embase (via Ovid) and Persian databases including SID and Magiran for articles published until November 2019 with language restrictions (English or Persian) but no time restrictions. The risk of bias in the studies was assessed using the Cochrane guidelines. The results of the meta-analysis were reported as mean difference, and the heterogeneity of the studies was evaluated using I^2^. GRADE approach was used to assess the quality of the evidence.

**Results:**

Of the 528 reviewed articles, eight were eligible for this study and finally six studies were included in the meta-analysis. According to the meta-analysis conducted on 128 preterm infants, the mean gastric residual volume (MD = − 2.11; 95% CI: − 2.76 to − 1.45, *P* < 0.00001) and mean frequency of vomiting (MD = − 0.84; 95% CI: − 1.37 to − 0.31; *P* = 0.002) were significantly lower in the massage therapy group compared to the control group. The mean abdominal circumference (MD = − 1.51; 95% CI: − 4.86 to 1.84; *P* = 0.38) and mean gastric residual number (MD = − 0.05; 95% CI: − 0.34 to 0.24; *P* = 0.74) were lower in the massage therapy group compared to the control group, although not in a statistically significant manner.

**Conclusion:**

Massage therapy significantly reduces the gastric residual volume and vomiting in preterm infants. Given the limited number of reviewed studies, the small number of neonates examined, and the short intervention periods, it is recommended that clinical trial be conducted with accurate methodology, longer interventions and larger sample sizes to ensure the effect of massage on feeding intolerance in these infants.

## Background

Premature birth is one of the most important direct causes of neonatal mortality, and the second leading cause of child mortality after pneumonia. Each year, one million out of the 15 million premature infants born die in the first year of life due to the complications of premature birth [1]. In recent years, significant improvements have been made in the care provided to these infants; however, prematurity is still a major cause of infant morbidity and mortality in developing countries [2].

Many premature babies are transferred to NICUs after birth and are fed by tube (nasal or oral route) [3]. Due to their prematurity, these infants lack a proper coordination between sucking, swallowing and breathing, and their risk of aspiration is therefore increased and they have to be fed by gavage [4]. Feeding problems are one of the factors causing the prolonged hospitalization of premature infants in NICUs [5]. The incomplete development of the gastrointestinal motility system in premature infants increases the gastric emptying time and decelerates bowel movements and eventually leads to symptoms such as constipation, abdominal distention and increased gastric residual volume (GRV) [6]. A high GRV increases the risk of gastrointestinal complications, such as necrotizing enterocolitis (NEC) [7]. Feeding intolerance is one of the major causes of weight loss in preterm infants that increases the risk of infection and prolongs prenatal feeding [3].

According to a meta-analysis conducted by Wang et al., massage therapy is a cost-effective intervention that improves weight gain and reduces hospitalization time in preterm infants [8]. In addition, massage therapy has a role in stimulating the digestive system and the vagus nerve in preterm infants and may thereby affect the growth of these infants [9].

Given the crucial role of neonatal nutrition and its association with cardiovascular disease risk factors, bone problems and cognitive function in adulthood [10] and the potential positive effect of massage on this variable [11], and also since some review studies [8, 12] have investigated the effect of massage therapy on a number of outcomes in preterm infants while no systematic review studies have yet examined the effect of this method on feeding intolerance in these infants, this review study was conducted to determine the effect of massage therapy on feeding intolerance in preterm infants.

## Methods

### Search strategy and study selection

This systematic review study examined randomized, controlled, clinical trials and quasi-experimental studies that evaluated the impact of massage therapy on preterm infants’ feeding intolerance. A search was carried out on all the articles published in English or Persian and indexed in English databases including Medline (via PubMed), Scopus, Cochrane Library, Google Scholar, Embase (via Ovid) and the Persian databases including SID and Magiran. The keywords used alone or in combination with other words included “preterm infants”, “feeding”, “feeding intolerance”, “massage”, “tactile-kinesthetic stimulation”, and their Persian equivalents.

All the articles reporting about randomized controlled clinical trials or quasi-experimental studies examining the effect of massage on the symptoms of feeding intolerance were reviewed. The PICO model (patient, intervention, comparison and outcome) was used. The patients included preterm infants (gestational age less than 37 full weeks). The intervention included the use of massage therapy. The comparison group consisted of those receiving routine care or any intervention other than massage therapy. Studies that investigated term infants or adults, and had no control groups were excluded.

### Primary outcomes

Mean GRV and number of gastric residual.

### Secondary outcomes

Mean frequency of vomiting and abdominal circumference.

### Data extraction

Two authors examined the acceptability and quality of the articles separately, and any disagreement was resolved by consensus and consultation with a third person. The following data were extracted and recorded in a checklist designed by the research team: first author’s name and year of publication, type of study, number of randomized participants, type of intervention and its duration, gestational age, follow-up time, outcomes and results (Table 1).

**Table 1:** Characteristics of included studies

Author	Design	Country	Sample size	Gestational age	Intervention	Comparison	Follow up	Outcomes	Outcome measures	Results
Tekgündüz et al., 2014 [16]	Quasi-experimental	Turkey	27	28 to 32 weeks	Abdominal massage 2 times daily for 5 days (each time = 15 min)	Routine Care	Last day	GRV, frequency of vomiting, abdominal circumference	Syringe, observation, meter	There was statistically significant difference between first day and last day in massage group.
Shaeri et al., 2017 [19]	RCT	Iran	64	29 to 33 weeks	Abdominal massage performed by a researcher 2 times daily for 5 days (each time = 15 min)	Routine Care	Last day	GRV, frequency of vomiting, abdominal circumference	Syringe, observation, meter	GRV, frequency of vomiting and abdominal circumference significantly reduced in the massage group, as compared the control group.
Mohamed & Ahmed, 2018 [15]	Quasi-experimental	Egypt	60	28 to 36 weeks	Abdominal massage performed 2 times daily for 5 days (each time = 15 min)	Routine Care	5th days of intervention	GRV, frequency of vomiting, abdominal circumference	Novel neonatal feeding intolerance tool.	There was statistically significant difference between first day and last day in massage group.
Ghasemi et al., 2019 [20]	RCT	Iran	28	Mean age was 32.4 weeks in intervention and 33 weeks in control groups	Abdominal massage performed by a trained physiotherapist 2 times daily for 5 days (each time = 15 min)	Routine Care	5th days of intervention	GRV	Syringe	GRV Significantly reduced in the massage group, as compared with the control group
Choi et al., 2015 [17]	Pilot study	South Korea	20	30 to 34 weeks	Massage (based on written protocol) performed 2 times daily for 14 days (each time = 15 min)	Routine Care	14th days of intervention	The number of gastric residual, abdominal circumference	GRV measured by a Syringe and the number of gastric residual recorded.	Frequency of gastric residual Significantly reduced in the massage group, as compared control group.
Fazli et al., 2017 [18]	RCT	Iran	34	30 to 34 weeks	Abdominal massage performed 2 times daily for 7 days (each time = 15 min)	Routine Care	“During the intervention days”	The number of gastric residual, frequency of vomiting	Vomiting and gastric residual recoded using nurses’ reports	Frequency of vomiting was significantly low in the massage group, but there was no significantly difference between two groups in terms of vomiting.
Karbandi et al., 2013 [13]	RCT	Iran	60	28 to 37 weeks	Passive massage movements performed daily in tree times for 5 days (each time = 15 min)	Routine Care	Last day	Feeding intolerance	Observations conducted by a researcher or nurses and recorded.	Feeding intolerance was significantly low in the massage group.
Fouda et al., 2018 [14]	Quasi-experimental	Egypt	60	< 37 week	Abdominal massage: 2 times daily for 7 days (each time = 15 min.	Routine Care	During 7 days	GRV, Vomiting.	Nutritional Assessment of Premature Neonate tool	In the end of 7th days, none of infants in both intervention groups had gastric residual and vomiting compared to the control group.

### Assessing the risk of bias in included studies

Allocation sequence bias, allocation concealment bias, participants, personnel and outcome assessor blinding bias, attrition bias and reporting bias were separately assessed by two authors using the Cochrane handbook. GRADE approach was used to assess the quality of the evidence.

### Statistical analysis

RevMan version 5.3 software was used to perform the meta-analysis and draw the risk of bias plots. The results of the meta-analysis were reported as mean difference with a confidence interval of 95%. The heterogeneity of the studies was evaluated using I^2^. The fixed effect was reported in the case of no significant heterogeneity (I^2^ ≤ 50%, *p* ≥ 0.01) and the random effect in the case of significant heterogeneity (I^2^ ≥ 50%, *p* ≤ 0.01). If the mean and standard deviation were reported zero in a study, its results were not entered into the meta-analysis due to the inability to enter these values ​​into RevMan software, and only the relevant values ​​were reported in the results.

## Results

**Fig. 1:**
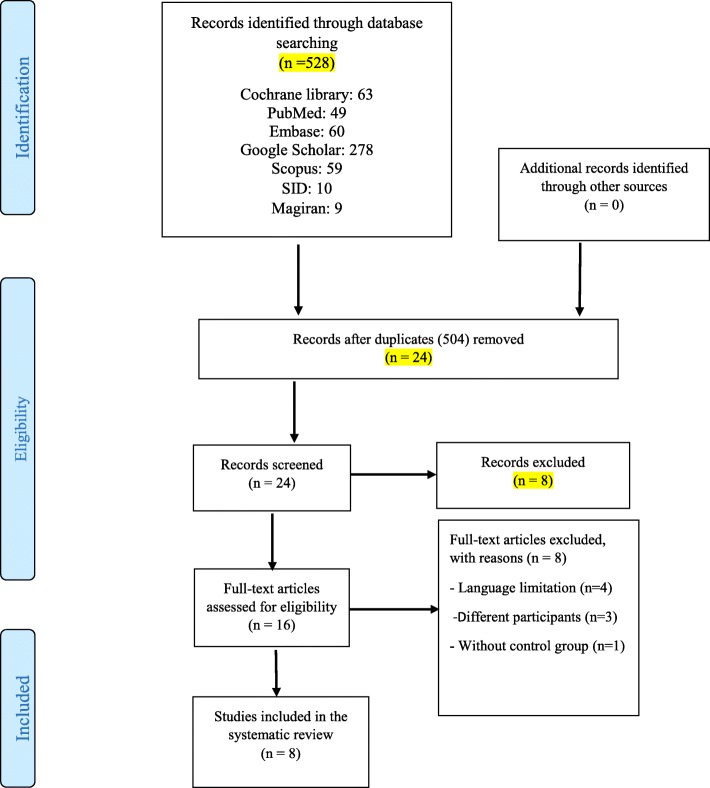
PRISMA flow diagram

Of the 528 articles searched, 504 were excluded for reasons such as non-relevance to the study subject, a non-interventional methodology and being a duplicate. Of the remaining 16 articles, four were excluded from the study for language restrictions, three for having different target groups and one due to the lack of a control group. Since the study by Karbandi et al. (13) reported the overall incidence or non-incidence of feeding intolerance rather than the symptoms of intolerance, only their overall results are presented in Table 1 and their data were not included in the meta-analysis. Also, in the study by Fouda et al. (14), the values ​​associated with the variables in question were zero, therefore, a meta-analytic assessment was not possible. Finally, six studies were included in the meta-analysis (Fig. 1).

### Characteristics of included studies

Two of the studies were conducted in Egypt [14, 15], one in Turkey [16], one in South Korea (17) and four in Iran [13, 18–20]. Three of the studies were quasi-experimental [14–16], one was a pilot study [17] and four were RCTs [13, 18–20]. The sample size of the preterm infants ranged from a minimum of 20 [17] to a maximum of 64 [19]. Massage therapy was performed in four of the studies twice per day for 5 days [15, 16, 19, 20], twice per day for 7 days in two other studies [14, 18], twice per day for 14 days in one study [17], and three times per day (each lasting 15 min) for 5 days in another study [13]. The type of massage applied in the included studies was abdominal massage in six of the studies [14, 15, 18–20], massage based on written protocol in one study [17] and passive massage movements in another study [13]. GRV was measured using a 5-cc syringe, abdominal circumference was measured using a meter, and the mean frequency of vomiting was estimated by observation and recorded in a checklist (Table 1). “The acceptance criteria of feeding tolerance included the presence of the gastric residuals of less than half of the previous meal, no vomiting, and lack of distention” [18].

### Bias in the studies

**Fig. 2:**
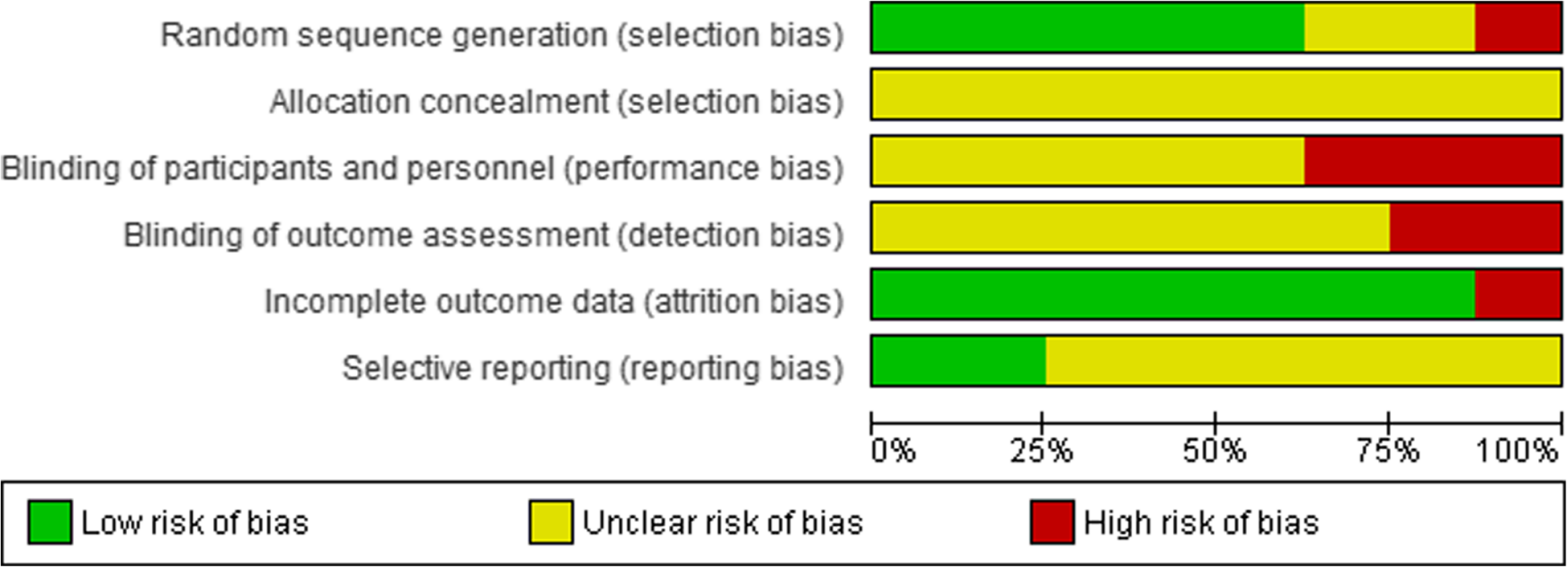
The risk of bias graph

**Fig. 3:**
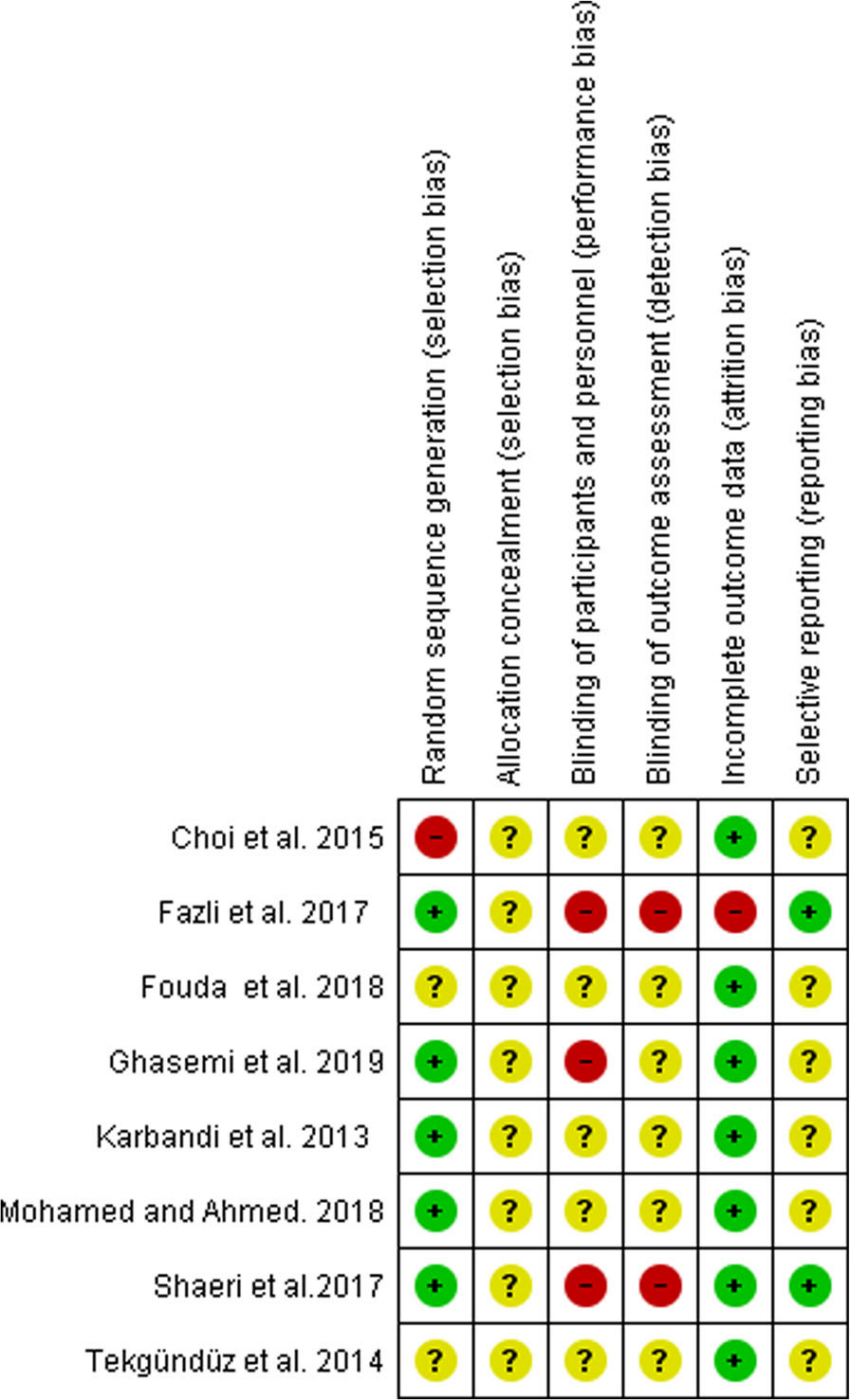
The risk of bias summary

The allocation sequence bias had a low risk in six of the studies [13–15, 18–20], an unclear risk in one study [16] and a high risk in the other [17]. The allocation concealment bias had an unclear risk in all the studies [13–20]. The participants, personnel and outcome assessor blinding bias had an unclear risk in six of the studies [13–17, 20] and a high risk in two [18, 19]. The incomplete outcome bias had a high risk in only one study [18] and a low risk in the others. The selective reporting bias had an unclear risk in six studies [13–17, 20] and a low risk in two others [18, 19] (Figs. 2 and 3, Table 2). The results of GRADE approach for evidence are reported in Table 3.

**Table 2:** Risk of bias summary in included studies

Bias risk	Tekgündüz et al., 2014 [16]	Shaeri et al., 2017 [19]	Mohamed & Ahmed, 2018 [15]	Ghasemi et al., 2019 [20]	Choi et al., 2015 [17]	Fazli et al., 2017 [18]	Karbandi et al., 2013 [13]	Fouda et al., 2018 [14]
Random sequence generation (selection bias)	**?**	Y	Y	Y	N	Y	Y	Y
Allocation concealment	?	?	?	?	?	?	?	?
Blinding of participant and personnel	?	N	?	?	?	N	?	?
Blinding of outcome assessment	?	N	?	?	?	N	?	?
Incomplete outcome data	Y	Y	Y	Y	Y	N	Y	Y
Selective reporting	Y	Y	?	?	?	Y	?	?

(?): Unclear risk of bias; Yes (Low risk); N: No (High risk)

**Table 3:** Quality assessment of included studies based on GRADE approach

No. of studies	Design	Risk of bias	Inconsistency	Indirectness	Imprecision	Other considerations	Abdominal massage	Routine Care	Pooled effect Relative (95% CI)	Final judgment
GRV										
3	Randomized trials	No Serious	No Serious	No serious indirectness	No serious imprecision	No serious	17.18/76	19.97/76	−2.11 [−2.78, −1.45]	⦻⦻⦻⦻ High
Number of gastric residual										
2	Randomized trials	No Serious	No Serious	No serious indirectness	Serious imprecision^b^	No serious	0.32/25	0.35/24	−0.05 [− 0.34, 0.24]	⦻⦻⦻◯ Moderate
Vomiting frequency										
2	Randomized trials	No Serious	Very serious inconsistency^a^	No serious indirectness	No serious imprecision	No serious	0.16/44	1.17/43	−0.84 [−1.37, − 0.31]	⦻⦻◯◯ Low
Abdominal circumference										
4	Randomized trials	No Serious	Very serious inconsistency^a^	No serious indirectness	Serious imprecision^b^	No serious	24.53/86	26.30/85	−1.51 [−4.86, 1.84]	⦻◯◯◯ Very low

^a^ I^2^ is higher than 40%, ^b^ Not met optimal information size/ CI is very wide

## Meta-analysis results

### GRV

**Fig. 4:**
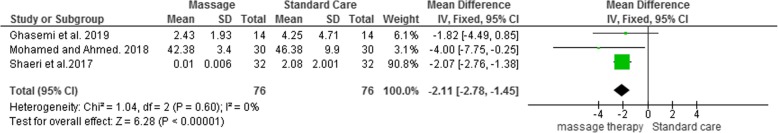
Forest plot for estimate the effect of massage on the GRV in the preterm infants

The mean GRV was significantly lower in the massage group than that in the standard care group (MD = − 2.11; 95% CI: − 2.76 to − 1.45; *P* = 0.60; I^2^ = 0%; df = 2; Chi^2^ = 1.04, *P* < 0.00001), (Fig. 4).

In the study by Tekgündüz et al. [16], the mean and standard deviation of GRV were 4.91 ± 3.21 in the massage therapy group on the first day and 0.00 ± 0.00 on the last day, which shows a significant reduction (*P* < 0.05); in the control group, however, GRV was 2.6 ± 1.15 on the first day and 0.00 ± 0.00 on the last day, which does not show a significant difference with the first day (*P* > 0.05).

In the study conducted by Fouda et al. [14] on 60 preterm infants, the first intervention group (*n* = 20) received massage therapy one hour before feeding the infant, the second intervention group (n = 20) received massage therapy one hour after feeding, and the control group (n = 20) received no massage. In the control group, on the seventh day of the intervention, two infants (10%) had a GRV less than 25%, 13 (65%) had 25 to 50% of GRV and five (25%) had more than 50% of GRV, while in both intervention groups, GRV was zero; since the variables with values ​​of zero cannot be entered into RevMan software, these values were not included in the meta-analysis.

### The number of gastric residual

**Fig. 5:**
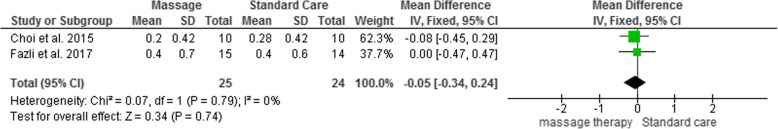
Forest plot for estimate the effect of massage on the number of gastric residual in the preterm infants

The mean number of gastric residual was lower in the massage group than in the standard care group, although not significantly (MD = − 0.05; 95% CI: − 0.34 to 0.24; *P* = 0.79; I^2^ = 0%; df = 1; Chi^2^ = 0.07, *P* = 0.74), (Fig. 5).

### Mean frequency of vomiting

**Fig. 6:**
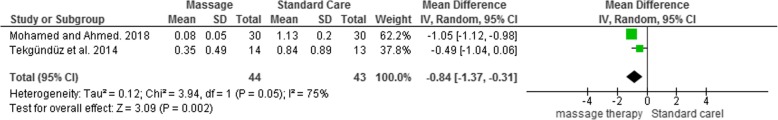
Forest plot for estimate the effect of massage on the vomiting frequency in the preterm infants

The mean frequency of vomiting was significantly lower in the massage group than that in the standard care group (MD = − 0.84; 95% CI: − 1.37 to − 0.31; *P* = 0.002) and the heterogeneity level was high (I^2^ = 75%; Tau2 = 0.12; Chi2 = 3.94, *P* = 0.05) (Fig. 6).

In the study by Ghasemian et al. [20], the mean and standard deviation of vomiting frequency during the 5 days of the intervention was 0.27 ± 0.07 in the control group, while no vomiting was reported in the intervention group. Also, in the study by Shaeri et al. [19], the mean and standard deviation of vomiting frequency in the post-intervention stage was 0.133 ± 0.01 in the control group and 0.00 ± 0.00 in the intervention group, which suggests a significant reduction in the intervention group compared to before the intervention (*P* < 0.001). According to the aforementioned reasons, the mean frequency of vomiting was not meta-analyzed in these two studies.

### Abdominal circumference

**Fig. 7:**
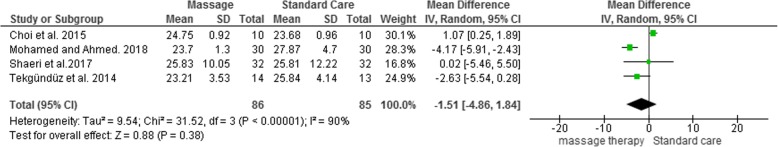
Forest plot for estimate the effect of massage on the abdominal circumference in the preterm infants

The mean abdominal circumference was lower in the massage group than that in the standard care group although not in a statistically significant manner (MD = − 1.51; 95% CI: − 4.86 to 1.84; *P* = 0.38), and the level of heterogeneity was high (I^2^ = 90%; Tau^2^ = 9.54; Chi^2^ = 31.52, *P* < 0.0001), (Fig. 7).

## Discussion

In the present meta-analysis, massage therapy reduced the mean GRV, mean frequency of vomiting, mean abdominal circumference and mean gastric residual number in preterm infants, but this reduction was not statistically significant for the mean gastric residual number and abdominal circumference.

In preterm infants, feeding tolerance depends on the maturation rate of the gastrointestinal tract (hormone and enzyme function, bacterial colonization, etc.), most of which occurs in the last 20 weeks of gestation. Nonetheless, an increase in microvilli that increases the absorption area continues in the last trimester of pregnancy and afterwards [21]. In premature infants, bacterial colonization is abnormal for several reasons, such as cesarean delivery, widespread use of antibiotics and NICU hospitalization, which makes them vulnerable to feeding intolerance and NEC [21–24].

Interventions to prevent or treat feeding intolerance should be designed to support or resolve the problems associated with inadequate gastrointestinal tract maturation, such as problems with motility and digestion [25]. Parasympathetic nerve stimulation is one of the mechanisms that may contribute to the positive effect of massage on feeding intolerance. Massage stimulates the gastrointestinal tract by stimulating the parasympathetic nerve, thereby increasing gastrointestinal motility and making food easier to digest [26].

Measuring the GRV is one of the best ways to detect gastric emptying delays [27]. In a clinical trial with massage therapy (two times per day for 3 days, 20 min each time) performed for patients in ICU, Momenfar et al. reported that massage reduced GRV significantly in the intervention group compared to the control group (*P* < 0.001), [28]. The results of their study are in line with the findings of the present meta-analysis, but with the difference that the majority of the interventions in the present study had lasted for at least 5 days, whereas in Momenfar’s study, the intervention duration was 3 days.

In another clinical trial, Uysal et al. [29] concluded that massaging patients hospitalized in the neurosurgery ward reduces GRV, vomiting and flatulence (abdominal circumference) significantly. The results of this study are in agreement with the present review in terms of vomiting and GRV but inconsistent in terms of abdominal circumference. In a study by Warren [30], massaging adult patients also reduced GRV and abdominal circumference, which is inconsistent with the present meta-analysis with regard to abdominal circumference. In a study by Choi et al. [17], although massaging increased defecation frequency significantly, the abdominal circumference of the infants did not show a significant difference on the 14th day of the massage therapy.

An increased GRV raises the likelihood of vomiting and consequently the risk of aspiration. Preventing an increase in GRV and vomiting frequency is therefore very important in patients with enteral nutrition [31]. In a clinical trial performed by Babai et al. [32] on 70 preterm infants hospitalized at the NICU (35 patients per group), the prophylactic administration of oral erythromycin at a dose of 2.5 mg/kg of bw for six hours over 10 days caused a significant reduction in GRV in the intervention group; however, there were no significant differences in vomiting frequency between the two groups. The prophylactic administration of erythromycin has side-effects such as hypertrophic pyloric stenosis; meanwhile [33], according to a review study by Vickers [12], no adverse effects have been reported for massage. Massage therapy has other positive effects on premature infants as well, such as strengthening the immune system, promoting development, reducing stress, increasing pain tolerance and reducing hospitalization time [34]. In addition to the benefits noted for premature infants, massage therapy also has positive effects on their mothers, as it reduces the symptoms of depression and anxiety in them [35] and promotes the mother-infant interaction [36].

### Strengths and limitations of the study

The main strengths of this review included the consideration of preterm infants as a vulnerable group, the use of non-pharmacological methods and the low risk of incomplete outcome bias (attrition) in more than 70% of the reviewed studied.

None of the studies provided any information on allocation concealment; also, the risk of personnel and outcome assessor blinding bias was unclear or high in most studies, which meant lower-quality studies. Based on the GRADE approach for the comparison of the massage group and the routine care group, the available evidence regarding GRV, number of gastric residual, vomiting frequency and abdominal circumference were high, moderate, low and very low in terms of quality, respectively. Therefore, the results of the vomiting frequency and abdominal circumference are close to real with little confidence. Another limitation of this review was restriction of language to Persian and English. Also, low sample size and differences in the duration and frequency of massage in included studies were other limitations that may contribute to the high heterogeneity in the meta-analyses of abdominal circumference and number of gastric residual outcomes.

## Conclusion

Massage therapy significantly reduces GRV and vomiting in preterm infants. Given the limited number of reviewed studies and the small number of neonates examined and the shorter intervention periods, clinical trials with accurate methodology, longer interventions and larger sample sizes are needed to ensure the effect of massage on feeding intolerance in these infants. Moreover, given that all the reviewed studies were from countries in Asia, the impact of this intervention is recommended to be examined on feeding intolerance in European countries.

## Data Availability

Data sharing not applicable to this article as no datasets were generated or analysed during the present study.

## Abbreviations

NICUs: Neonatal Intensive Care Units
GRADE: Grading of Recommendations Assessment; Development and Evaluation
MD: Mean Difference
GRV: Gastric Residual Volume
NEC: Necrotizing Entero Colitis
RCT: Randomized Controlled Trial
ICU: Intensive care unit
BW: Body Weight
